# Efficacy analysis of compartmentalization for ambient CH_4_ activation mediated by a Rh^II^ metalloradical in a nanowire array electrode†

**DOI:** 10.1039/d0sc05700b

**Published:** 2020-12-08

**Authors:** Benjamin S. Natinsky, Brandon J. Jolly, David M. Dumas, Chong Liu

**Affiliations:** Department of Chemistry and Biochemistry, University of California Los Angeles California 90095 USA chongliu@chem.ucla.edu; California NanoSystems Institute (CNSI), University of California Los Angeles CA 90095 USA

## Abstract

Compartmentalization is a viable approach for ensuring the turnover of a solution cascade reaction with ephemeral intermediates, which may otherwise deactivate in the bulk solution. In biochemistry or enzyme-relevant cascade reactions, extensive models have been constructed to quantitatively analyze the efficacy of compartmentalization. Nonetheless, the application of compartmentalization and its quantitative analysis in non-biochemical reactions is seldom performed, leaving much uncertainty about whether compartmentalization remains effective for non-biochemical reactions, such as organometallic, cascade reactions. Here, we report our exemplary efficacy analysis of compartmentalization in our previously reported cascade reaction for ambient CH_4_-to-CH_3_OH conversion, mediated by an O_2_-deactivated Rh^II^ metalloradical with O_2_ as the terminal oxidant in a Si nanowire array electrode. We experimentally identified and quantified the key reaction intermediates, including the Rh^II^ metalloradical and reactive oxygen species (ROS) from O_2_. Based on such findings, we experimentally determined that the nanowire array enables about 81% of the generated ephemeral intermediate Rh^II^ metalloradical in air, to be utilized towards CH_3_OH formation, which is 0% in a homogeneous solution. Such an experimentally determined value was satisfactorily consistent with the results from our semi-quantitative kinetic model. The consistency suggests that the reported CH_4_-to-CH_3_OH conversion surprisingly possesses minimal unforeseen side reactions, and is favorably efficient as a compartmentalized cascade reaction. Our quantitative evaluation of the reaction efficacy offers design insights and caveats into application of nanomaterials to achieve spatially controlled organometallic cascade reactions.

## Abstract 2

We integrated theory with experiment to evaluate the catalytic cycle of seemingly incompatible steps enabled by nanowire array for CH_4_-to-CH_3_OH conversion, and determined the array’s efficacy in the context of microscopic compartmentalization.

## Graphical abstract

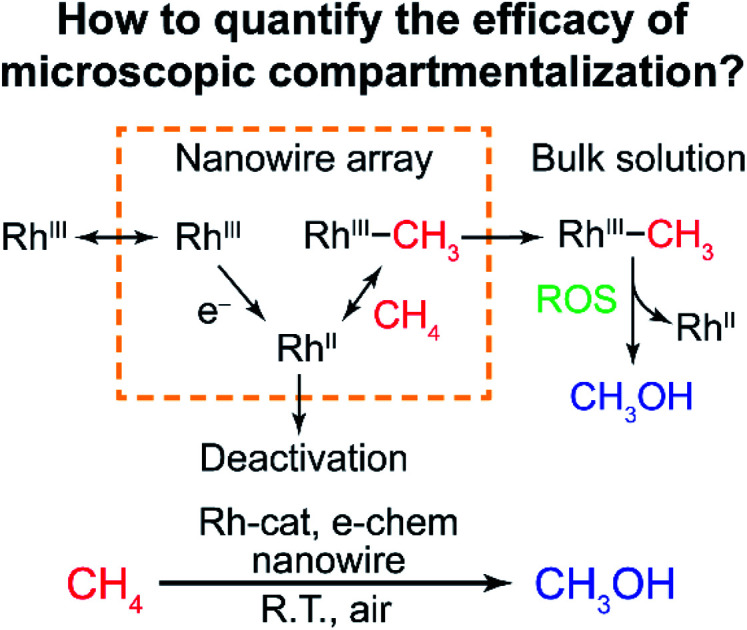

## Introduction

**Fig. 1:**
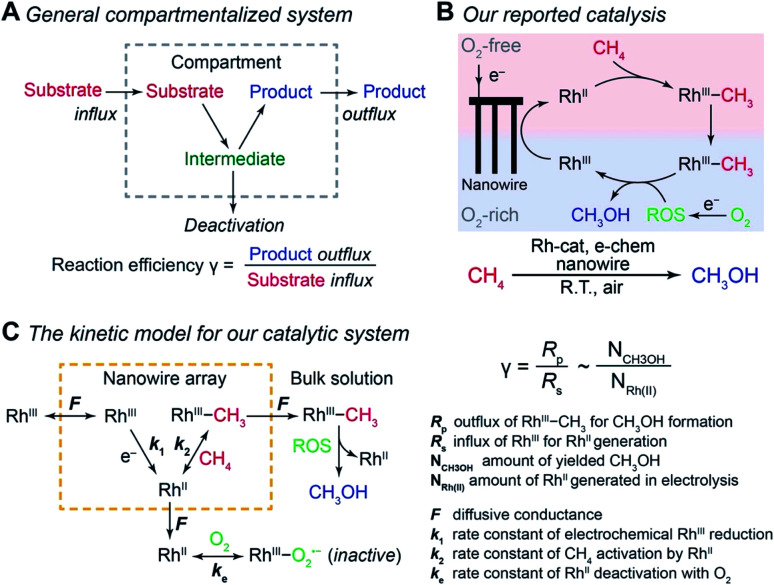
Reaction efficiency *γ* in compartmentalized cascade reactions. (A) A generalized schematic for the compartmentalization cascade. (B) The proposed mechanism of our previously reported system for ambient CH_4_-to-CH_3_OH conversion.^20^ Rh^III^, rhodium(iii) tetramesityl porphyrin iodide; Rh^II^, rhodium(ii) tetramesityl porphyrin metalloradical; Rh^III^–CH_3_, methylated rhodium(iii) tetramesityl porphyrin; ROS, reactive oxygen species; Rh-cat, rhodium(iii) tetramesityl porphyrin iodide; e-chem, electrochemistry; R.T., room temperature. (C) A theoretical framework of a kinetic model for the CH_4_-to-CH_3_OH system in the context of a compartmentalized cascade. A detailed mathematical derivation of *γ* is available in Section 1B of the ESI.†

Compartmentalization, ubiquitous in biology, allows efficient transfer of reaction intermediates or ephemeral species within a multienzyme cascade reaction in an intracellular medium.^1–3^ By segregating subsequent catalytic sites from the bulk environment at the microscopic or even nanoscopic level, spatial control of catalytic reactions ensures the functionality of biological metabolism in a factory-like manner with high efficiency.^4–6^ Here the key to a successful compartmentalized cascade reaction is the capability of confining a transient intermediate within the compartment and preventing its outflux that leads to either intermediate loss or deactivation (Fig. 1A). A number of theoretical models have been established to evaluate the capability of compartmentalized cascade reactions in fulfilling this task,^7,8^ predominantly in the context of enzymatic catalytic reactions.^9–13^ Reaction efficiency (*γ*), defined as the ratio between the product outflux and the substrate influx to the compartment for a one-to-one stoichiometric reaction (Fig. 1A), quantitatively measures the efficacy of a cascade system in retaining and utilizing the intermediate species generated within the compartment.^6,9,14,15^ With the value of *γ* commonly approaching unity for cascade reactions in biology,^4–6,14^ nature masters the design strategy of compartmentalization for enzymatic reactions.

The benefits of confining catalytic reactions spatially at microscopic and nanoscopic levels in nature inspire the development of other non-enzymatic cascades, mostly with surface-attached molecular, nanoparticle, or enzyme catalysts in porous media such as metal–organic frameworks^16–18^ or superposed on scaffolds.^10–12^ Nonetheless, analyzing and determining the value of *γ*, a quantitative figure-of-merit for the efficacy of compartmentalization, remains infrequent, to say the least, for non-enzymatic scenarios. As reported in recent publications,^9,16,19^ the scarcity of experimentally determined *γ* values casts uncertainty about the effectiveness of a certain design strategy of compartmentalization. A synergistic study comparing experimentally determined *γ* values with the one from a theoretical model will offer valuable insights whether the designed cascade reaction is effective without undesirable side effects, ascertain the merits of designed compartmentalization, and further justify the design strategy of non-enzymatic compartmentalized systems. This research report aims to offer an exemplary case of such a study for organometallic catalysis with compartmentalized systems.

We are interested in employing microscopic concentration gradients within nanomaterials to design organometallic catalytic cycles of seemingly incompatible steps, in which key reaction intermediates will be quickly deactivated once they diffuse out of the nanomaterials. In our previous report,^20^ a cascade catalysis with O_2_-sensitive reaction intermediates was established to achieve ambient CH_4_-to-CH_3_OH conversion with O_2_ as the terminal oxidant assisted by electricity. As shown in Fig. 1B, the nanowire array electrode electrochemically reduces rhodium(iii) tetramesityl porphyrin iodide (Rh^III^) into rhodium(ii) tetramesityl porphyrin metalloradical (Rh^II^) in aprotic solvent 1,2-difluorobenzene (1,2-DFB). At the same time, the nanowire array reduces O_2_ into reactive oxygen species (ROS) and creates a sharp O_2_ concentration gradient in a local O_2_-free environment near the bottom of the nanowire array under aerobic conditions. In the O_2_-free microenvironment, two equivalents of the generated Rh^II^ species, while highly reactive with O_2_^21^, activate one equivalent of CH_4_ ambiently to yield methylated rhodium(iii) tetramesityl porphyrin (Rh^III^–CH_3_) and rhodium(iii) tetramesityl porphyrin hydride (Rh^III^–H). Both species are proposed to react with the generated ROS that remain to be further identified (*vide infra*), leading to the formation of CH_3_OH. While no barrier is present to physically separate the liquid phase within the nanowire array from the bulk solution, the whole system can be considered to be a compartmentalized cascade with the nanowire array as the compartment with its unique microenvironment (Fig. 1C), analogous to the previous argument in the case of two-enzyme cascades in which enzymes are placed in close proximity at the nano-scale.^12,22^ The overall process was reported to be catalytic, achieving a turnover number up to 52 000 within 24 hours.^20^ The synergy between nanomaterials and organometallic chemistry warrants a new catalytic route of CH_4_ functionalization, while additional studies are needed to understand the underlying mechanism and quantitatively evaluate the efficacy of the strategy that interfaces nanowires with organometallics.^23^

Here we report our analysis of the above-mentioned CH_4_-to-CH_3_OH catalysis in the context of a compartmentalized cascade. We translated the reported catalytic system into a theoretical model that estimates the numerical value of *γ*. Electron paramagnetic resonance (EPR) spectroscopy with a spin-trap agent, along with other characterization experiments, unraveled that the predominant ROS present in the system is a superoxide and illustrated its role in CH_3_OH formation. This piece of mechanistic insight allowed us to subsequently determine the value of *γ* in the catalysis, which surprisingly amounts to more than 80%. The high value of measured *γ* is consistent with our theoretical framework and illustrates the efficacy of the created nanoscopic concentration gradient with minimal side reactions. Our results demonstrate that carefully designed compartmentalization, spatially controlling the occurrence of organometallic reactions in solution at the microscopic length scale, can circumvent undesirable reactions efficiently and create organometallic catalytic cycles impossible in homogeneous solutions.

## Results and discussion

### A theoretical framework of cascade reactions in a nanowire array electrode

Generally applicable for non-enzymatic cascade design as noted above, one question that we ask is whether our previously reported design employing the nanowire array and a concentration gradient is effective in utilizing the ephemeral O_2_-sensitive intermediate, Rh^II^, whose activation of CH_4_ was considered turnover-limiting.^20,24^ Our approach to this question is to construct a numerical model in the context of compartmentalization and analyze the reaction efficiency *γ* that will be valuable for future design consideration. Fig. 1C shows the reported reaction in the context of compartmentalized cascade reactions. Here the compartment, schematically shown as a yellow-colored dashed box, is defined as the O_2_-free liquid phase within the nanowire array where Rh^II^-initiated CH_4_ activation is proposed to take place. Following its definition,^9,15^*γ* is expressed as the ratio between the outward flux of Rh^III^–CH_3_ for CH_3_OH formation (*R*_p_) and the rate of Rh^II^ generation (*R*_s_) during the electrolysis. Based on the proposed reaction mechanism (Fig. 1B and C), *R*_p_ and *R*_s_ are dictated by the kinetic rate constant of electrochemical reduction of Rh^III^ into Rh^II^ (*k*_1_) as well as the Rh^II^-initiated CH_4_ activation (*k*_2_), respectively. We note that *γ* could also be interpreted as the competition between the rate of CH_4_ activation (*R*_p_) in the compartment and the deactivation of Rh^II^ with O_2_ in the bulk, whose rate constant is denoted as *k*_e_. Because such a competition depends on the diffusive mass transport at a steady state, another important factor is the diffusive conductance *F*, as used in the design of enzymatic cascades,^6,9,13^ which describes the rate of mass transport for chemical species crossing into and out of the compartment boundary. However, in order to obtain the flux of a particular species in and out of the compartment, *F* must be normalized to the volume of the compartment *V* and Avogadro's number *N*_A_.^9^ We anticipate that *F* and *V* will be co-dependent, therefore we derive an expression for *F*/(*VN*_A_), denoted *F*_V_, in terms of the nanowire geometry (Section 1A of the ESI†). Since the Rh^II^, Rh^III^, and Rh^III^–CH_3_ species share the bulky metalloporphyrin structure, as a first-order approximation *F*_V_ is assumed to be the same among Rh^II^, Rh^III^, and Rh^III^–CH_3_, and only dependent on the morphology of the nanowire array. Assuming *k*_e_ → ∞ due to the reported rapid deactivation,^21^ we incorporated the above-mentioned components and derived a numerical model (Sections 1B of the ESI†), which describes *γ* in a compartmentalized cascade as shown below:1
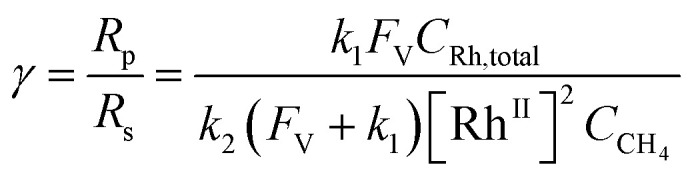
2
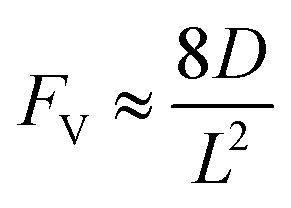
Here *C*_Rh,total_ is the total concentration of Rh species in the bulk solution, almost exclusively in the form of Rh^III^; [Rh^II^] is the steady-state concentration of Rh^II^ in the compartment during electrolysis that remains to be numerically calculated; *C*_CH_4__ is the concentration of CH_4_ in the bulk solution; *D* is the diffusion coefficient of the Rh species; and *L* is the length of the nanowire array. Similarly, we derived the expression of *γ* in the non-compartmentalized homogeneous solution (Section 1C of the ESI†). After solving the steady-state kinetic equations that include the mass transport across the compartment, we further derived the expressions of *γ* from eqn (1) for the nanowire array electrode:3
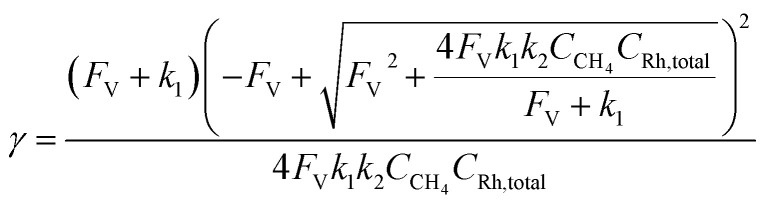
And the expressions of *γ* in a homogeneous solution:4
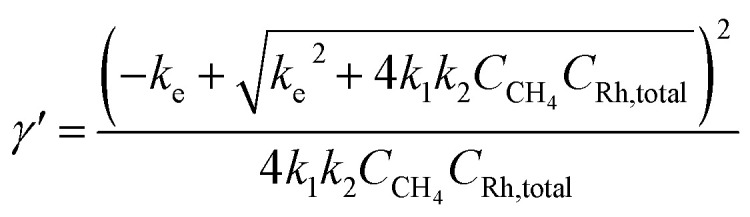
we note that *k*_e_ is explicitly incorporated in eqn (4) while we assumed *k*_e_ → ∞ in eqn (3). The above equations establish the theoretical framework for us to analyze the efficacy of the organometallic reactions in the nanowire array electrode.

The established theoretical model demands input from experimental results. Because of the high reactivity between Rh^II^ and O_2_ outside of the O_2_-free microenvironment in the nanowire array,^21^*i.e. k*_e_ → ∞, *γ* can be approximated as the ratio between the amount of generated CH_3_OH (*N*_CH_3_OH_) and Rh^II^ from electrochemical reduction of Rh^III^ (*N*_Rh(ii)_) (Fig. 1C), if we presume that the conversion from Rh^III^–CH_3_ to CH_3_OH is stoichiometric as corroborated by using our prior experimental evidence.^20^ While *N*_CH_3_OH_ is experimentally readily measurable as we have done before,^20^ the value of *N*_Rh(ii)_ is less accessible and requires the quantification of ROS because the electrochemical reduction of Rh^III^ into Rh^II^ is concurrent with the electrochemical reduction of O_2_ into the ROS (Fig. 1B). Therefore, identification and quantification of the ROS in this catalytic system is needed, not only for deeper insights about the chemical steps involved in the catalysis but also for a quantitative determination of *γ*.

### Identification and quantification of reactive oxygen species in the catalysis

The identity of the predominant ROS was probed by EPR spectroscopy with the addition of spin trap agent 5,5-dimethyl-1-pyrroline N-oxide (DMPO) during electrolysis.^25,26^ Superoxide (O_2_˙^−^), hydrogen peroxide (H_2_O_2_), and hydroxyl radical (˙OH) were presumed to be the possible ROS from O_2_ reduction in aprotic solvent systems.^27,28^ Among these possible ROS, H_2_O_2_, along with other hydroperoxide species, react with DMPO to yield the adduct DMPO–OH˙, which can be detected by EPR spectroscopy.^29^ While O_2_˙^−^ and ˙OH are short-lived,^27,30^ their reactions with DMPO yield more stable adducts DMPO–O_2_˙^−^ and DMPO–OH˙, whose prolonged lifetimes are roughly 1 and 20 minutes, respectively, at room temperature and much longer under liquid N_2_ conditions.^26^ By trapping the generated ROS with DMPO during the electrolysis and discerning the trapped radicals in EPR spectroscopy^26,31,32^ the possible presence of O_2_˙^−^ and H_2_O_2_/˙OH could be unveiled.

**Fig. 2:**
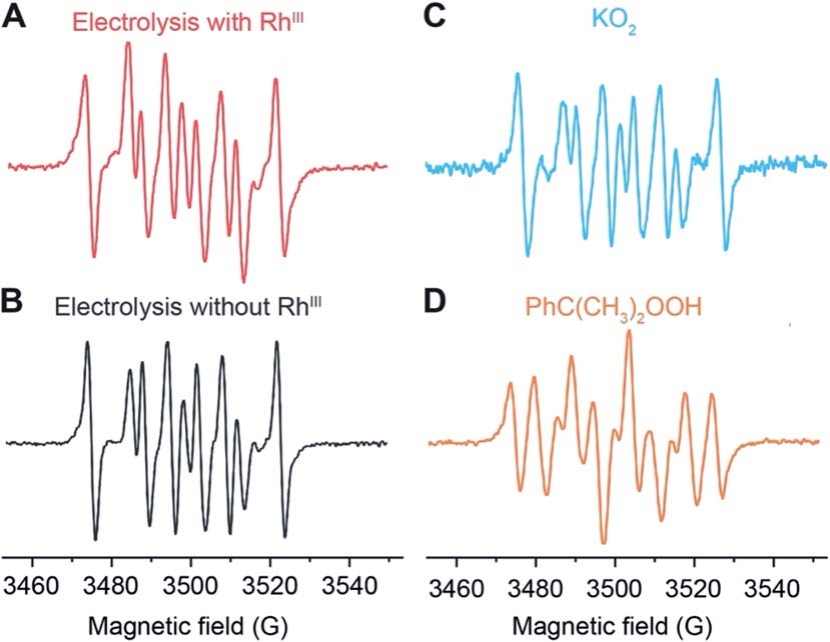
Electron paramagnetic resonance (EPR) spectra depicting the adducts formed in the reaction of 50 mM DMPO (5,5-dimethyl-1-pyrroline N-oxide): (A) the electrolysis with the Rh catalyst during CH_4_-to-CH_3_OH conversion; (B) control electrolysis in the absence of the Rh catalyst; (C) 0.5 mM potassium superoxide (KO_2_); (D) 0.5 mM 2-hydroperoxypropan-2-ylbenzene (PhC(CH_3_)_2_OOH, cumene hydroperoxide). Unless otherwise noted, the electrolyte solution is 0.1 M tetrabutylammonium perchlorate (TBAClO_4_) in 1,2-difluorobenzene (1,2-DFB). Following previously reported conditions,^20^ chronoamperometry was conducted at −1.4 V *vs.* SCE with the Si nanowire working electrode under a constant flow of a CH_4_/air mixture *P*_CH_4__/*P*_air_ = 35 in a customized electrochemical reactor (Fig. S1†) under ambient pressure.

EPR spectra indicate that O_2_˙^−^ is the predominant ROS during the CH_4_-to-CH_3_OH conversion. As reported in our previous work (Section 3A in the ESI†),^20^ CH_4_-to-CH_3_OH catalysis was conducted in a customized single-chamber electrochemical reactor (Fig. S1†), which was fed with a mixture of CH_4_ and air (*P*_CH4_/*P*_air_ = 35) at a constant flow rate at ambient pressure and room temperature. Chronoamperometry of −1.4 V *vs.* the Saturated Calomel Electrode (SCE) was performed in 1,2-DFB with 1 mM of Rh^III^ as the pre-catalyst and 0.1 M tetrabutylammonium perchlorate (TBAClO_4_) as the supporting electrolyte. A Si nanowire array with an average wire length of 15 μm and diameter of 50 nm (Fig. S2†), prepared based on the literature,^20,33^ was used as the working electrode with a Pt wire counter electrode and a Ag^+^/Ag pseudo-reference electrode equipped with a glass frit. 50 mM DMPO was added during the electrolysis to trap the generated ROS, and aliquots were taken for EPR experiments 5, 15, and 60 minutes after the addition of DMPO. Unless stated specifically, the aliquots were stored in liquid N_2_ before the EPR measurements, although the DMPO adduct has been observed to be stable at room temperature for at least 90 minutes (Fig. S3†). We note that the addition of DMPO *per se* does not significantly alter the electrochemistry in the system, if any, because previous studies have shown that DMPO is cathodically stable up to −2.35 V *vs.* SCE.^34^ The EPR spectrum from the aliquot 15 minutes after DMPO addition is shown in Fig. 2A, which is similar to the ones from the aliquot taken at 5 and 60 minutes after DMPO addition (Fig. S4†). This similarity suggests that the radical species observed in EPR spectroscopy is the predominant species in the steady state during the electrolysis and not all of the transient ROS involved in the catalytic cycle may be captured in our experiments. Control experiments include the DMPO-added electrolysis without the Rh^III^ pre-catalyst (Fig. 2B), the reaction between DMPO and 0.5 mM KO_2_ as a surrogate of O_2_˙^−^ (Fig. 2C and S5†), as well as the reaction between DMPO and 0.5 mM 2-hydroperoxypropan-2-ylbenzene (PhC(CH_3_)_2_OOH, cumene hydroperoxide) as a surrogate of H_2_O_2_/˙OH (Fig. 2D and S5†). The captured ROS during electrolysis is predominantly O_2_˙^−^, based on the similar spectra shown in Fig. 2A and C. Nonetheless, we note that the spectral pattern observed for the DMPO–O_2_˙^−^ in 0.1 M TBAClO_4_ in 1,2-DFB solution is noticeably distinct from the DMPO–OOH observed in the aqueous solvent.^25,26^ This difference can be rationalized by the employment of 1,2-DFB in place of H_2_O and the resulting solvation sphere surrounding the adduct, the reduced proton concentration, and the possibility of TBA^+^ cations to coordinate the anionic complex, which could explain the increased stability of the observed adduct upwards of 90 min. The similarity between Fig. 2A and B suggests that it is likely that the electrochemistry of Si nanowires rather than the Rh species is responsible for the generation of O_2_˙^−^.

**Fig. 3:**
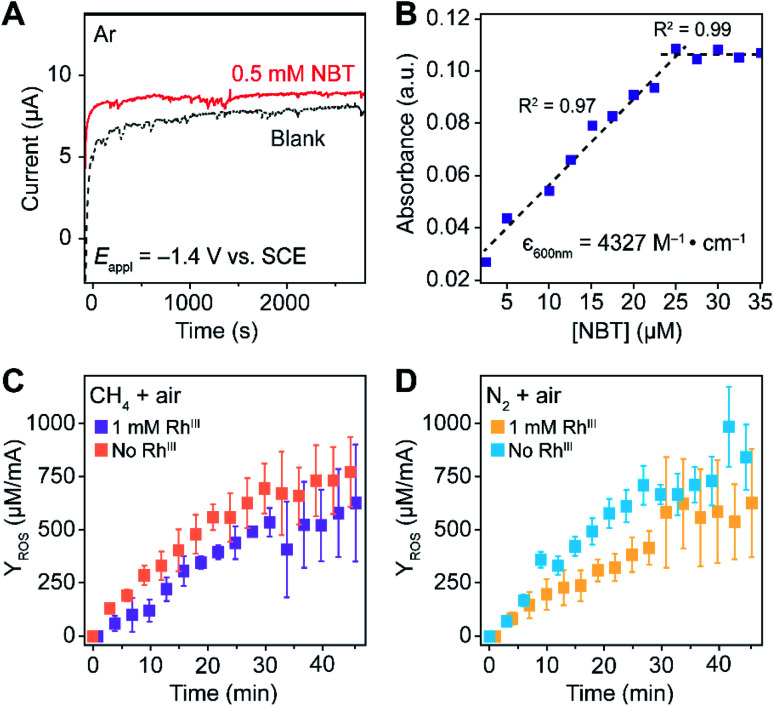
Quantification of superoxide (O_2_˙^−^) with O_2_˙^−^-selective chromogen nitroblue tetrazolium (NBT). (A) Chronoamperometry with a glassy carbon working electrode in Ar. (B) The absorbance at 600 nm in 1,2-DFB of a fixed concentration of KO_2_, a surrogate of ephemeral O_2_˙^−^, for varying concentrations of NBT. *ε*_600nm_, the established absorption coefficient at 600 nm of the yielded monoformazan from a stoichiometric reaction between NBT and O_2_˙^−^.^35^ (C and D) The yield of ROS (*Y*_ROS_), determined as the detected O_2_˙^−^ normalized by the average electric current, is displayed against electrolysis duration with a Si nanowire array of 15 μm length as the working electrode. (C) A CH_4_/air atmosphere (*P*_CH4_/*P*_air_ = 35) with (purple, *n* = 4) and without (orange, *n* = 3) 1 mM Rh^III^. (D) N_2_/air atmosphere (*P*_N2_/*P*_air_ = 35) with (yellow, *n* = 5) and without (blue, *n* = 5) 1 mM Rh^III^. −1.4 V *vs.* SCE 0.1 M TBAClO_4_ in 1,2-DFB. Error bars denote standard deviations.

We propose to quantify the generated ROS, predominantly O_2_˙^−^ at the steady state, by colorimetric assay with the use of nitroblue tetrazolium (NBT) chloride as a O_2_˙^−^-selective chromogen. NBT is reported to selectively react with the O_2_˙^−^ over H_2_O_2_ and other ROS,^35^ leading to the emergence of a purple color in monoformazan, with a maximum absorption peak at ∼530 nm,^35,36^ from a pale yellow background. The stoichiometric reactivity between NBT and O_2_˙^−^ (1 : 2), ensured by utilizing a concentration of NBT/O_2_˙^−^ < 2, enables the use of UV-Vis absorption spectroscopy for the quantification of O_2_˙^−^ generation.^35,37–39^ Additionally, NBT exhibits minimal reductive activity in organic solvent within its electrochemical window, which was not only reported in the previous literature^36^ but also shown in our cyclic voltammograms (Fig. S6†) and chronoamperometry (Fig. 3A) on a glassy carbon working electrode in 0.1 M TBAClO_4_ in 1,2-DFB solution. Thus, when added during the electrolysis under CH_4_-to-CH_3_OH conditions, NBT will have minimal interference with the O_2_-reducing electrode and act as an O_2_˙^−^ scavenger for a colorimetric quantification of the accumulated O_2_˙^−^. While multiple methods including fluorescence measurements are viable for the O_2_˙^−^ quantification,^38^ the absorbance at 600 nm from monoformazan after the reaction between NBT and O_2_˙^−^ (ref. ^36^) was chosen in order to mitigate interference from the optical absorbance and phosphorescence of Rh species (630–750 nm).^40^ Furthermore, due to the ephemeral nature of O_2_˙^−^ that hinders the preparation of a standard solution of known O_2_˙^−^ concentration,^35,41^ we specifically designed experiments that establish a calibration curve of the absorbance at 600 nm which accounts for the stoichiometric reaction between NBT and KO_2_, the O_2_˙^−^ surrogate.^35^ When an increasing concentration of NBT was mixed with a fixed concentration of KO_2_ in 1,2-DFB, the absorbance at 600 nm follows a linear correlation before plateauing (Fig. 3B), illustrating a stoichiometric reaction between NBT and O_2_˙^−^ without other chromogenic side reactions. This led us to determine the absorption coefficient at 600 nm of the yielded monoformazan, *ε*_600 nm_ = 4327 M^−1^ cm^−1^ (*R*^2^ = 0.97) (Section 3B of the ESI†). A similar linear response between NBT and O_2_˙^−^ was also observed in the presence of Rh catalysts in 1,2-DFB (Fig. S7†), which suggests that the presence of Rh^III^ in the bulk solution does not interfere with the colorimetric assay.

The rate of O_2_˙^−^ generation was quantified with the use of NBT in the nanowire-based cascade system for CH_4_-to-CH_3_OH conversion. Under the same electrochemical conditions with the use of the Si nanowire array electrode of 15 μm length at −1.4 V *vs.* SCE, aliquots of the electrolyte solution were sequentially sampled, measured for optical absorbance at 600 nm, and applied to calculate the amount of accumulated O_2_˙^−^ (Section 3C of the ESI†). The yields of the net accumulated O_2_˙^−^ (*Y*_ROS_), normalized by the average electric current during electrolysis, were shown as a function of electrolysis duration in CH_4_/air and N_2_/air atmospheres (*P*_CH_4__/*P*_air_ and *P*_N_2__/*P*_air_ = 35; Fig. 3C and D, respectively). The initial slopes of the *Y*_ROS_ before plateauing (∂*Y*_ROS_/∂*t*) were determined as the rate of electrochemical ROS generation in the compartmentalized cascade reaction. ∂*Y*_ROS_/∂*t* = 18 ± 4 and 22 ± 4 μM mA^−1^˙min^−1^ in the CH_4_/O_2_ atmosphere with the presence and absence of 1 mM Rh^III^ (*n* = 4 and *n* = 3; purple and orange traces in Fig. 3C, respectively); ∂*Y*_ROS_/∂*t* = 16 ± 4 and 23 ± 3 μM mA^−1^˙min^−1^ in the N_2_/air with the presence and absence of 1 mM Rh^III^ (*n* = 5; yellow and blue traces in Fig. 3D, respectively). The similar values of ∂*Y*_ROS_/∂*t* between the CH_4_ and N_2_ atmosphere suggests that the ROS formation is independent of the gaseous environment. The similar values of ∂*Y*_ROS_/∂*t* with and without Rh^III^ suggests that the electrode surface of Si nanowires is primarily responsible for ROS generation, albeit the presence of Rh^III^ does seem to lower the ROS yield. Here we note that ∂*Y*_ROS_/∂*t* could be underestimated, because NBT is less reactive towards other ROS that may be concurrently generated during the electrochemical process.

### Experimental determination and analysis of reaction efficiency γ

Our successful quantification of the electrochemical ROS generation rate (∂*Y*_ROS_/∂*t*) led to an experimentally determined value of reaction efficiency *γ*. As the reductive current is responsible for the generation of both ROS and CH_4_-activating Rh^II^, the equation for *γ* in Fig. 1C can be written as:5
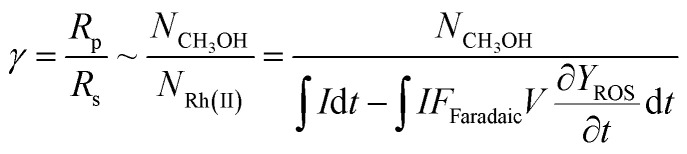
Here *I* denotes the electric current, *F*_faradaic_ the faradaic constant, and *V* the volume of electrochemical reactor. Eqn (5) leads to *γ* = 81% for a 3 h electrolysis of CH_4_ activation (Section 1D of the ESI†), based on the results of our previous report^20^ and the value of *Y*_ROS_ determined in Fig. 3. The experimentally determined value of *γ* is relatively close to unity, suggesting that a large portion of the generated Rh^II^ activates CH_4_ before diffusing out the nanowire array despite the high reactivity between Rh^II^ and O_2_ (Fig. 1C).

**Fig. 4:**
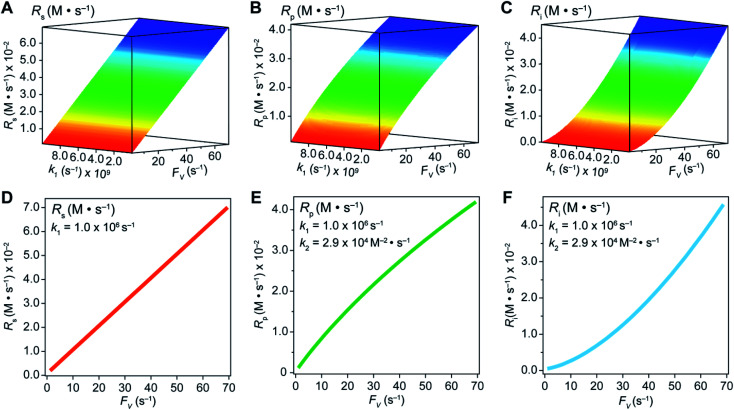
Graphical representations of substrate conversion (*R*_s_), product formation (*R*_p_), and intermediate outflux (*R*_i_) in the compartmentalized system. Panels (A), (B), and (C) represent *R*_s_, *R*_p_, and *R*_i_, respectively, as a function of both *F*_V_ and *k*_1_. Panels (D), (E), and (F) represent *R*_s_, *R*_p_, and *R*_i_, respectively, as a function of *F*_V_ for a constant value of *k*_1_ (1.0 ×10^6^ s^−1^). *k*_2_ was experimentally determined to be 2.9 × 10^4^ M^−2^ s^−1^ within the nanowire array in our previous report.^20^

In an effort to compare our experimentally determined *γ* values with the theoretical maximum, we numerically calculated the values of *γ* based on the established theoretical framework for a nanowire-based compartmentalization. Following the model displayed in Fig. 1C, the calculated values of *R*_s_ (Fig. 4A and D), *R*_p_ (Fig. 4B and E), and the Rh^II^ flux diffusing out of the compartment *R*_i_ (Fig. 4C and F) were displayed as a function of *k*_1_ and *F*_V_ (Section 1B of the ESI†). These calculations use *k*_e_ → ∞, *C*_CH_4__ = 9.5 mM,^20^*C*_Rh,total_ = 1.0 mM, and *k*_2_ = 2.9 × 10^4^ M^−2^ s^−1^ for Rh^II^-initiated CH_4_ activation within the nanowire array as determined in our previous report.^20^ Because the rate of electrochemical reduction of Rh^III^ (*k*_1_) was not readily determinable in our system, *k*_1_ was assigned as a range of values spanning four orders of magnitude (10^6^ to 10^10^ s^−1^) based on a typical range reported for electron transfer of metalloporphyrin complexes in the literature.^42^ Nonetheless, Fig. 4 shows that the effect of compartmentalization is not sensitive to the value of *k*_1_, evident as the *k*_1_ term cancels out in eqn (S14), (S18), and (S19)† during the derivation as long as *k*_1_ is sufficiently larger (≥10^6^ s^−1^) than the value of *F*_V_, with an increased *F*_V_ value leading to increased fluxes both outward and inward of the compartment. Because the value of *F*_V_ is dependent on the nanowire array's morphology, such a trend suggests that we can control and possibly optimize the mass transport across the compartment by controlling the length *L* of the nanowire array based on eqn (2).

**Fig. 5:**
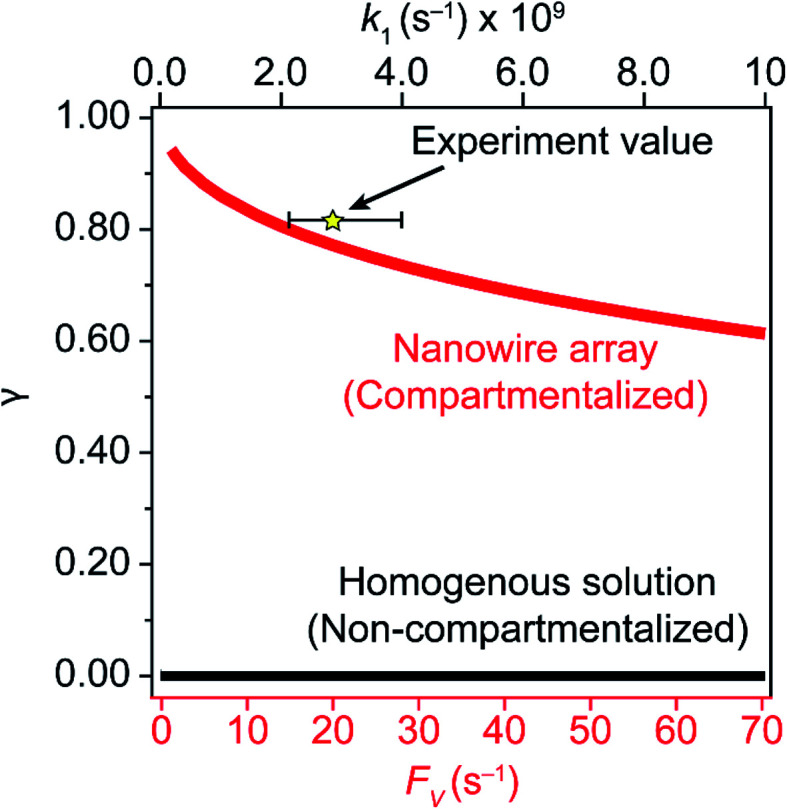
Reaction efficiency *γ* plotted as a function of diffusive conductance *F*_V_ for the scenarios with nanowire-enabled compartmentalization (red trace) and *γ* plotted as a function of *k*_1_ without nanowire-enabled compartmentalization (black trace). The experimentally determined data point of *γ* for nanowire array of 15 μm length is represented by the star. The error bar presents the approximation when deriving the experimental value of *F*_V_ (Section 1A in the ESI†).

A comparison between the experimental value of *γ* with simulation results suggests that the Rh-based organometallic catalysis enabled by the nanowire array is indeed functioning near its theoretical limit. Given the strong dependence on the value of *F*_V_ for *R*_s_, *R*_p_, and *R*_i_, we envisioned that *γ* is mostly a function of *F*_V_, whose relationship is displayed as the red trace in Fig. 5. While *R*_s_, *R*_p_, and *R*_i_ all increase with larger values of *F*_V_ (*vide supra*), overall a larger *F*_V_ value tends to decrease the value of *γ* (Fig. 5). Such a trend is corroborated with our previous observation that a nanowire array of longer length *L*, hence smaller *F*_V_ value based on eqn (2), corresponds to a larger yield of CH_3_OH before mass transport becomes rate-limiting.^20^ One thing to note is that our developed theoretical framework does not account for those Rh^III^ molecules that enter and leave the nanowire compartment without undergoing the initial electrochemical reduction reaction. However, the alignment of the experimental and theoretical values indicates that this phenomenon, while it cannot be excluded completely, remains minimal in our system. In comparison, the value of *γ* for a non-compartmentalized scenario, *i.e.* homogeneous solution without a nanostructured electrode, is also shown as the black trace in Fig. 5 (Section 1C in the ESI†). A clear difference is observable as the value of *γ* for the non-compartmentalized scenario is virtually near zero (at most 0.001% indeed), in line with our previous experimental work that utilized a planar electrode surface and resulted in no CH_3_OH generation.^20^ We also positioned our experimentally determined *γ* value in Fig. 5, after we determined *F*_V_ = 20. s^−1^ given *L* = 15 μm and *D* = 5.6 × 10^−10^ m^2^ s^−1^ in 1,2-DFB as measured before.^20^ The error bar in Fig. 5 represents the approximation incurred when deriving the expression for *F*_V_ (eqn (2)) (Section 1A in the ESI†). A good agreement between the experimental and theoretical values of *γ* for the nanocompartment was observed (Fig. 5), yet the slightly higher value of experimentally derived *γ* might originate from the underestimation of ∂*Y*_ROS_/∂*t* (*vide supra*). The agreement indicates that minimal unforeseen side reactions, if any, are present in the catalyst system and the proposed benefit of a nanowire-generated O_2_-free microenvironment for Rh^II^-initiated CH_4_ activation is well demonstrated.

Despite the findings that O_2_˙^−^ is the dominant ROS at steady state, we propose that O_2_˙^−^ is not the immediate oxidant that reacts with Rh^III^–CH_3_ for CH_3_OH formation. We found that under strictly dry conditions, no CH_3_OH was observed in a mixture of KO_2_ and the as-synthesized Rh^III^–CH_3_. Yet a stoichiometric amount of CH_3_OH, calculated using ^1^H NMR, was observed in experiments with Rh^III^–CH_3_ and KO_2_ “wet” in 1,2-DFB (5.1 ± 0.3 mM H_2_O based on Karl Fischer titration). Along the same lines, 1 : 1 reactivity was observed upon mixing Rh^III^–CH_3_ with hydroperoxide species such as cumene hydroperoxide or *t*-butyl hydroperoxide in dry 1,2-DFB (Section 4 of the ESI†).^20^ Such observations prompted us to propose that trace hydroperoxide, namely H_2_O_2_, is constantly generated during the electrolysis and is the immediate reactant towards Rh^III^–CH_3_ to afford CH_3_OH as the product. We speculate that the electrochemical reduction of O_2_ to O_2_˙^−^ is followed by a chemical protonation step to generate other ROS such as a hydroperoxyl radical (HO_2_˙),^43^ which is susceptible to heterolytic and homolytic cleavages and eventually converges on H_2_O_2_.^44–46^ Whether the conversion of HO_2_˙ to H_2_O_2_ proceeds electrochemically or chemically should be dependent on the proton donor concentration in solution and the reduction potential applied.^46^ Once the hydroperoxide species is formed, CH_3_OH formation proceeds from its reaction with Rh^III^–CH_3_. Such mechanistic consideration would be useful in designing other ROS-initiated CH_4_-activation reactions.

Additional design insights are available from our results. We examined the dependence of *γ* on different hypothetical values of the CH_4_-activation rate constant *k*_2_. Smaller values of *k*_2_ significantly lower *γ*, *i.e.* the efficiency of compartmentalization created by the nanowire array (Fig. S8†). Such a trend is reasonable since a lower value of *k*_2_ will lead to more prominent outward flux *R*_i_ of the unreacted Rh^II^ species which will introduce a larger percentage of undesirable Rh^II^ deactivation. Therefore, while our Rh-based catalysis demonstrates the benefits of compartmentalization by constructing a catalytic cycle of seemingly incompatible steps for organometallics, we caution that the efficacy of this strategy depends on the specific chemical system under consideration. As the value of *γ* depends on *F*_V_ and thus the nanostructure's morphology, such as nanowire length *L* in our case, there exists an optimal morphology of nanostructures for specific organometallic compounds' reactivities in order to create an effective microenvironment and efficient cascade reactions with minimal detrimental deactivation. A quantitative evaluation of reaction efficiency *γ*, however estimated, is recommended in order to justify the introduction of nanostructures and a micro-environment. A general numerical design framework of nanostructures for a typical organometallic catalytic cycle that includes oxidative addition and reductive elimination is currently being developed in the authors' laboratory.

## Conclusion

In this work, we applied the concept of reaction efficiency *γ* in biochemical cascades to quantitatively evaluate the efficacy of compartmentalization for organometallic reactions with the use of nanowire array electrodes. A high *γ* value approaching unity, the theoretical limit, was experimentally observed, suggesting minimal detrimental side reactions. This observation indicates that with suitable design it is possible to employ nanomaterials to spatially control organometallic reactions and achieve efficient cascade with ephemeral intermediates, analogous to the biological counterparts with near-unity *γ* values. This work quantitatively highlights the transformative power of spatial control at the nanoscale for new chemical reactivity.

## Author's contribution

C. L. supervised the project. C. L. and B. S. N. designed experiments and wrote the paper. B. S. N. synthesized the compounds and conducted electrochemical characterization experiments with assistance from D. M. D. B. J. J. performed the mathematical derivations on the compartmentalized system. All the authors discussed the results and assisted during the manuscript preparation.

## Conflicts of interest

The authors declare no competing financial interest.

## Supplementary Material

SC-012-D0SC05700B-s001
