# Supplementary material for: Efficacy analysis of compartmentalization for ambient CH4 activation mediated by a RhII metalloradical in a nanowire array electrode
Source: Chem Sci. 2020 Dec 8;12(5):1818–25. doi: 10.1039/d0sc05700b (PMC8179293; doi:10.1039/d0sc05700b)
Supplement: SC-012-D0SC05700B-s001 [file SC-012-D0SC05700B-s001.pdf]

Electronic Supplementary Material (ESI) for Chemical Science.
This journal is © The Royal Society of Chemistry 2020

Supplementary Information

Efficacy analysis of compartmentalization for ambient CH_4 activation mediated by Rh^{II} metalloradical in nanowire array electrode

Benjamin S. Natinsky[1†], Brandon J. Jolly[1†], David M. Dumas[1], Chong Liu[1,2*]

[1] Department of Chemistry and Biochemistry, University of California, Los Angeles, California 90095, United States

[2] California NanoSystems Institute (CNSI), University of California, Los Angeles, Los Angeles, CA 90095, USA

[†] Equal contributions of authorship

* To whom correspondence may be addressed. Email: chongliu@chem.ucla.edu

Table of Contents

Materials and Methods

Section 1. Derivation of the theoretical framework S3

Section 2. Chemicals and methods S8

Section 3. Identification and quantification of oxygen reduction species S9

Section 4. Reactivity between Rh^{III}–CH_3 and ROS S11

Supplementary Figures

Figure S1. Photograph of a customized electrochemical reactor used for bulk electrolysis S12

Figure S2. Scanning electron microscopy image of silicon nanowire array S13

Figure S3. Electron paramagnetic resonance spectra of DMPO + KO_2 after 90 min S14

Figure S4. Electron paramagnetic resonance spectra of DMPO + KO_2 at varying sampling times S15

Figure S5. Electron paramagnetic resonance spectra of DMPO + KO_2 and DMPO + cumene hydroperoxide S16

Figure S6. Cyclic voltammograms of NBT on a glassy carbon electrode under argon S17

Figure S7. UV-Vis absorption spectrum and Beer's Law curve of NBT + $O_2^{\cdot-}$ reaction product (monoformazan) S18

Figure S8. Derived traces of γ as a function of F_V with varying k_2 values S19

Additional references S20

Section 1. Derivation of the theoretical framework

A. Derivation of diffusive conductance (F). We adapt a definition for diffusive conductance (F) where F is proportional to the compartment permeability (p), compartment surface area (SA), and Avogadro's number (N_A), to suit the nanowire array geometry.[1-2] Permeability of the anaerobic nanowire-based compartment is defined here in terms of the diffusion coefficient (D) and diffusion path length (Δx), where Δx is approximated as being half of the nanowire length, under the assumption that a particular species will traverse, on average, half the length of the nanowire.

$$p \approx \frac{D}{\Delta x} \tag{S1}$$

Where $\Delta x = 0.5 \times L$. We note that such an assumption of Δx is an approximate and may incur sizable errors in the calculation of F values (vide infra). Yet, this approximation is experimentalized supported by our previous work, which indicates that the bottom half of the 15 μm wire array is mostly O_2-free.[3]

We then multiplied p by SA and N_A to obtain:

$$F \approx \frac{D \times SA \times N_A}{0.5 \times L} \tag{S2}$$

However, in a previous report[1-2], F is normalized to $V \times N_A$ and then multiplied by concentration in order to obtain the flux of particular species. We postulate that within our nanowire array, F and V will be related, therefore we derive a term for $F/(VN_A)$ as F_V in the unit of s^{-1}. In addition, we derive the volume of the anaerobic compartment as $0.5 \times SA$ multiplied by the length of the compartment, $L - \Delta x = 0.5 \times L$, to obtain the following:

$$F_V = \frac{F}{VN_A} \approx \frac{\frac{D \times SA \times N_A}{0.5 \times L}}{0.5 \times SA \times (L - \Delta x) \times N_A} \tag{S3}$$

$$F_V = \frac{F}{VN_A} \approx \frac{8D}{L^2} \tag{S4, Eqn 2 in main text}$$

Furthermore, since all relevant Rh porphyrin species are about the same size, the diffusion coefficient D for the substrate, intermediate, and product (Rh^{III}, Rh^{II}, and Rh^{III}–CH_3, respectively), were approximated as being equal and was previously found for Rh^{III} to be 5.62×10^{-10} m^2 s^{-1}.[3]

Lastly, we estimate an error of ±1 μm to Δx in the derivation of $F/(VN_A)$ for the 15 μm wire array experimental reaction efficiency (star with error bar in Figure 5). This estimation accounts for variances in the location of the anaerobic region established within the nanowire array compartment, which is evident in our experimental quantification of the locally O_2-free environment in previous report.[3] A more in depth approximation of F is currently being explored, and will be discussed in future work.

B. Derivation of reaction efficiency γ for nanowire-based compartmentalization. The enclosed reactions within the nanowire domain are represented by rate constants, k, as well as the diffusive conductance, F_V as presented in Figure 1C. As Rh^{III} enters the system based on the diffusive conductance, F_V, it is reduced to the active Rh^{II} governed by the kinetic rate of electron transfer, k_1. Then, the rate constant for CH_4 activation within the wire array domain is denoted as k_2, which was previously quantified as 2.9×10^4 $L^2 \cdot mol^{-2} \cdot s^{-1}$.[3] However, the reaction of Rh^{II} with CH_4 is contingent on its ability to outcompete diffusion and subsequent elimination with O_2 outside the compartment, k_e, creating the inactive superoxo adduct $Rh^{III}–O_2^{\bullet-}$.[4] Here, k_e is assumed to be infinitely large ($k_e \rightarrow \infty$) such that Rh^{II} in the bulk $[Rh^{II}]_b$ is considered negligible ($[Rh^{II}]_b = 0$). In this system, Rh^{III} is defined as the substrate, Rh^{II} as the intermediate species, and $Rh^{III}–CH_3$ the product. Within the model, $C_{Rh,total}$ is the total concentration of Rh species in the bulk solution, almost exclusively in the form of Rh^{III}; C_{CH_4} is the concentration of CH_4 in the bulk solution; $[Rh^{III}]$, $[Rh^{II}]$, and $[Rh^{III} - CH_3]$ are the steady-state concentrations of Rh^{III} that influxes into the compartment, Rh^{II} generated from electroreduction of Rh^{III} within the compartment, and $Rh^{III}–CH_3$ generated upon CH_4 activation, respectively. Thus, the concentration of each species in the compartment is outlined by equations S5–8 below:

$$\frac{d[Rh^{III}]}{dt} = F_V(C_{Rh,total} - [Rh^{III}]) - k_1[Rh^{III}] \quad \text{(S5)}$$

$$\frac{d[Rh^{II}]}{dt} = k_1[Rh^{III}] - k_2[Rh^{II}]^2 C_{CH_4} - F_V([Rh^{II}] - [Rh^{II}]_b) \quad \text{(S6)}$$

$$\frac{d[Rh^{III} - CH_3]}{dt} = k_2[Rh^{II}]^2 C_{CH_4} - F_V[Rh^{III} - CH_3] \quad \text{(S7)}$$

$$\frac{d[Rh^{II}]_b}{dt} = F_V([Rh^{II}] - [Rh^{II}]_b) - k_e[Rh^{II}]_b \quad \text{(S8)}$$

$[Rh^{II}]$ may be solved for in terms of $C_{Rh,total}$ when we assuming $[Rh^{II}]_b = 0$,

$$F_V(C_{Rh,total} - [Rh^{III}]) - k_1[Rh^{III}] = 0 \quad \text{(S9)}$$

$$F_V C_{Rh,total} = (F_V + k_1)[Rh^{III}] \quad \text{(S10)}$$

$$[Rh^{III}] = \frac{F_V C_{Rh,total}}{F_V + k_1} \quad \text{(S11)}$$

Solving for $[Rh^{II}]$ from Equation S6 at steady state leads to the expression of $[Rh^{II}]$:

$$k_2[Rh^{II}]^2 C_{CH_4} + F_V([Rh^{II}] - [Rh^{II}]_b) - k_1[Rh^{III}] = 0 \quad \text{(S12)}$$

$$[Rh^{II}] = \frac{-F_V + \sqrt{{F_V}^2 + \frac{4F_V k_1 k_2 C_{CH_4} C_{Rh,total}}{F_V + k_1}}}{2k_2 C_{CH_4}} \quad \text{(S13)}$$

The expression of $[Rh^{II}]$ leads us to derive the mathematical expression of substrate conversion (R_s), product formation (R_p), and intermediate outflux (R_i) in the compartmentalized system:

$$R_s = k_1[Rh^{III}] = \frac{k_1 F_V C_{Rh,total}}{F_V + k_1} \quad \text{(S14)}$$

$$R_i = F_V([Rh^{II}] - [Rh^{II}]_b) \quad \text{(S15)}$$

$$R_p = k_2[Rh^{II}]^2 C_{CH_4} \quad \text{(S16)}$$

$$\gamma = \frac{R_p}{R_s} = \frac{k_1 F_V C_{Rh,total}}{k_2(F_V + k_1)[Rh^{II}]^2 C_{CH_4}} \quad \text{(S17, Eqn 1 in main text)}$$

Assuming $[Rh^{II}]_b = 0$, R_s is invariant and remains the same as Equation S14. Therefore,

$$R_i = \frac{-{F_V}^2 + F_V\sqrt{{F_V}^2 + \frac{4F_V k_1 k_2 C_{CH_4} C_{Rh,total}}{F_V + k_1}}}{2k_2 C_{CH_4}} \quad \text{(S18)}$$

$$R_p = \frac{\left(-F_V + \sqrt{{F_V}^2 + \frac{4F_V k_1 k_2 C_{CH_4} C_{Rh,total}}{F_V + k_1}}\right)^2}{4k_2 C_{CH_4}} \quad \text{(S19)}$$

$$\gamma = \frac{(F_V + k_1)\left(-F_V + \sqrt{{F_V}^2 + \frac{4F_V k_1 k_2 C_{CH_4} C_{Rh,total}}{F_V + k_1}}\right)^2}{4F_V k_1 k_2 C_{CH_4} C_{Rh,total}} \quad \text{(S20, Eqn 3 in main text)}$$

C. Derivation of reaction efficiency γ for non-compartmentalized homogenous solution. Similar calculations in previous sessions can be applied to the non-compartmentalized scenarios in homogeneous solutions. While we assuming $k_e \rightarrow \infty$ in previous sections, here k_e is explicitly incorporated.

$$\frac{d[Rh^{III}]}{dt} = -k_1 C_{Rh,total} \quad \text{(S21)}$$

$$\frac{d[Rh^{II}]}{dt} = k_1 C_{Rh,total} - k_2 [Rh^{II}]^2 C_{CH_4} - k_e [Rh^{II}] \quad \text{(S22)}$$

$$\frac{d[Rh^{III} - CH_3]}{dt} = k_2 [Rh^{II}]^2 C_{CH_4} \quad \text{(S23)}$$

Solving for $[Rh^{II}]$ from Equation S12 at steady state:

$$k_2 [Rh^{II}]^2 C_{CH_4} + k_e [Rh^{II}] - k_1 C_{Rh,total} = 0 \quad \text{(S24)}$$

$$[Rh^{II}] = \frac{-k_e + \sqrt{{k_e}^2 + 4k_1 k_2 C_{CH_4} C_{Rh,total}}}{2k_2 C_{CH_4}} \quad \text{(S25)}$$

Here, the substrate conversion (R_s'), product formation (R_p'), and intermediate generation (R_i') in the homogeneous solution are expressed as:

$$R_s' = k_1 C_{Rh,total} \quad \text{(S26)}$$

$$R_i' = k_e [Rh^{II}] \quad \text{(S27)}$$

$$R_p' = k_2 [Rh^{II}]^2 C_{CH_4} \quad \text{(S28)}$$

Taking the expression of $[Rh^{II}]$ leads to the expression of reaction efficiency γ' in homogeneous solution,

$$R_i = \frac{-k_e^{\ 2} + k_e\sqrt{k_e^{\ 2} + 4k_1k_2C_{CH_4}C_{Rh,total}}}{2k_2C_{CH_4}} \tag{S29}$$

$$R_p = \frac{\left(-k_e + \sqrt{k_e^{\ 2} + 4k_1k_2C_{CH_4}C_{Rh,total}}\right)^2}{4k_2C_{CH_4}} \tag{S30}$$

$$\gamma' = \frac{\left(-k_e + \sqrt{k_e^2 + 4k_1k_2C_{CH_4}C_{Rh,total}}\right)^2}{4k_1k_2C_{CH_4}C_{Rh,total}} \quad \text{(S31, Eqn 4 in main text)}$$

D. Calculation of reaction efficiency in nanowire array compartment.

The experimental calculation of the reaction efficiency γ can be written as:

$$\gamma = \frac{R_p}{R_s} \sim \frac{N_{CH3OH}}{N_{Rh(II)}} = \frac{N_{CH3OH}}{\int Idt - \int IF_{Faradaic}V\frac{\partial Y_{ROS}}{\partial t}dt} \quad \text{(S32, Eqn 5 in main text)}$$

Here, N_{CH3OH} and $N_{Rh(II)}$ denote the amount of generated CH_3OH and Rh^{II} from electrochemical reduction during the reaction, respectively; I denotes the electric current, $F_{Faradaic}$ the Faradaic constant, V the volume of electrochemical reactor, and $\partial Y_{ROS}/\partial t$ the electrochemical ROS generation rate that can be experimentally determined. The amount of Rh^{II} generated throughout a 3 h electrolysis was determined by the difference in the amount of charge passed (in Coulombs) with and without the pre-catalyst in solution. Under the aforementioned electrolysis conditions with $P_{CH4}/P_{air} = 35$, the total charged passed with and without Rh^{III} in solution was 0.63 C and 0.30 C, respectively. The increase in charge in the presence of Rh^{III} was attributed to the reduction of Rh^{III} to Rh^{II}, which was determined as 0.33 C. This is equivalent to 13.64 µmoles Rh^{II} generated in a 3 h electrolysis. This was converted to Rh^{III}–CH_3 via the reaction stoichiometry of 2 equivalents of Rh^{II} per 1 equivalent of CH_4. Thus, the theoretical maximum CH_3OH synthesized was 6.82 µmoles. This quantity was compared alongside the observed average amount of CH_3OH obtained previously, 5.55 µmoles[3], which resulted in a reaction efficiency of 81 %.

Section 2. Chemicals and methods

A. Chemicals. The commercial reagents used in the various procedures were purchased from Sigma Aldrich, VWR and Fisher Chemicals unless otherwise noted; all chemicals were used as received unless specified. The deionized (DI) water that was used throughout the experiments came from a Millipore Milli-Q Water Purification System. The protonated tetramesityl porphyrin ligand, (TMP)H_2, was purchased from Frontier Scientific. Nitroblue tetrazolium (NBT) chloride salt and 5,5-Dimethyl-1-pyrroline N-oxide (DMPO) were purchased from Cayman Chemical. 1,2-difluorobenzene (1,2-DFB) was purchased from Oakwood Chemical. The 1,2-DFB used in the glove box was distilled from CaH_2 and freeze-pump-thawed to remove residual O_2. The 1,2-DFB used in other experiments outside of the glove box was purified with activated 4 Å molecular sieves to remove residual moisture and methanol (CH_3OH) impurities. Tetrabutylammonium perchlorate ($TBAClO_4$), purified by recrystallization in ethanol (C_2H_5OH), was used as the electrolyte in all electrochemical measurements.

B. Chemical and materials characterizations. Electron paramagnetic resonance (EPR) spectra were recorded on a X-band continuous wave (CW) spectrometer equipped with a 14” Bruker EPR magnet. The X-band CW microwave bridge is composed of a high sensitivity cavity (4119HS-W1). Ultraviolet-visible (UV-Vis) absorption spectroscopy was conducted on an Agilent Technologies Cary 60 spectrometer. Spectra of one-dimensional proton nuclear magnetic resonance (^1H NMR) were recorded on a Bruker AV300 (300 MHz) spectrometer. Chemical shifts for protons are reported in parts per million (ppm) and deuterated benzene (C_6D_6) from Cambridge Isotope Laboratories was used as the locking solvent. A gas chromatograph equipped with a mass spectrometer (GC-MS, Agilent Technologies 5975 with Inert XL Selective Detector) was used for CH_3OH determination and quantification. A Mettler Toledo C20 Coulometric Karl Fischer (KF) Titrator was utilized for the determination of water content in neat 1,2-DFB.

C. (TMP)Rh−I (Rh^{III}) and (TMP)Rh−CH_3 (Rh^{III}−CH_3). Both syntheses follow procedures published by Wayland et al[5] and were utilized in past work published by our group.[3]

D. Si wire array. Si nanowire array applied in the bulk electrolysis experiments was prepared following a modified electroless etching recipe based on the works by Huang et al[6] and was utilized in past work published by our group.[3]

Section 3. Identification and quantification of oxygen reduction species

A. Spin trap studies for reactive oxygen species detection. DMPO was dissolved in the electrolyte solution, 0.1 M $TBAClO_4$ in 1,2-DFB, to a final concentration of 200 mM and was stored at –30 °C. 3 mg of KO_2 and 15 mg of 18-crown-6 (2:3 equivalents) were dissolved in 20 mL of the electrolyte solution. This solution was diluted 4-fold and stored under argon (Ar) at –30 °C. 500 μL of the DMPO stock solution was mixed with the KO_2 stock solution, while in a tandem experiment it was mixed with cumene hydroperoxide (Figure 2). Additionally, both KO_2 and cumene hydroperoxide were mixed together in the same vial (Figure S5). In all experiments, the concentration of DMPO was 50 mM; moreover, the concentrations of KO_2 and cumene hydroperoxide were either 500 μM or 1 mM. When DMPO was combined with KO_2, cumene hydroperoxide, or both, the samples were mixed vigorously and immediately frozen under liquid N_2. They were transported frozen to the EPR facility located at the California Institute of Technology where spectra were obtained at room temperature. For observation of the electrochemically generated reactive oxygen species, typical bulk electrolysis experiments were conducted using a Gamry Instruments Interface 1000-E potentiostat with a customized electrochemical cell (Figure S1) which contained 0.1 M $TBAClO_4$ in 1,2-DFB. In the cell was the silicon (Si) nanowire array working electrode (with an average wire length of 15 μm and diameter of 50 nm) (Figure S2), Pt wire counter electrode, and a Ag^+/Ag pseudo-reference electrode with a glass frit. A mixture of CH_4 (Airgas, 99.5%) and house air were introduced into the reactor at a fixed ratio of 35 ($P_{CH4}/P_{air} = 35$) under a constant flow rate with the use of mass flow controllers (Omega Engineering, Inc., Model: FMA5502A 0-10 sccm). The bulk electrolysis was conducted under a constant E_{appl} of –1.4 V vs. Saturated Calomel Electrode (SCE). Upon stabilization of the current, DMPO was added into the electrochemical chamber to a final concentration of 50 mM. In a single experiment, samples were taken 5, 15, and 60 min after the addition of DMPO and were immediately frozen under liquid N_2. The samples were transported to the EPR facility under liquid N_2 and EPR spectra were taken at room temperature (Figure S4). Additionally, the thawed reaction mixtures were subjected to room temperature conditions for up to 90 min after which a subsequent spectrum was taken (Figure S3).

B. Determining the extinction coefficient of monoformazan generated between NBT and $O_2^{•-}$. The extinction coefficient of monoformazan generated between NBT and $O_2^{•-}$ at 600 nm was determined following a similar protocol reported before.[7] 3 mg of KO_2 and 15 mg of 18-crown-6

(2:3 equivalents) were dissolved in 20 mL of the electrolyte solution. This solution was diluted 4-fold and stored under argon (Ar) at –30 °C. 100 μM NBT, 100 μM Rh^{III}, and varying concentrations of the stock KO_2 solution were mixed in the electrolyte solution, 0.1 M $TBAClO_4$ in 1,2-DFB, and transferred to a quartz cuvette for UV-Vis absorption measurements (Figure S7). The absorbance at 600 nm was chosen and used for future experiments as to not interfere with the characteristic absorption and phosphorescence emission of Rh^{III} (Figure S7). The extinction coefficient of NBT in 1,2-DFB at 600 nm, ε_{600nm}, was identified in order to quantify the superoxide ($O_2^{\bullet-}$) concentration. 500 μL of the KO_2 stock solution was mixed with varying concentrations of NBT and diluted with the electrolyte solution to a final volume of 2 mL. The reaction between KO_2 and NBT was allowed to progress overnight to ensure completion and then UV-Vis absorption spectra were obtained. The ε_{600nm} was found to be 4327 $M^{-1}\cdot cm^{-1}$ (Figure 3B).

C. Constant potential electrolysis with NBT for $O_2^{\bullet-}$ quantification. All electrochemical tests were performed using a Gamry Instruments Interface 1000-E potentiostat. In an Ar-filled glovebox, cyclic voltammetry (CV) (Figure S6) and bulk electrolysis experiments (Figure 3A) were performed on a 500 μM NBT solution in a glass vial equipped with a Teflon top fitted with electrode and gas inlet/outlet ports. The cell contained 0.1 M $TBAClO_4$ in 1,2-DFB with a Pt wire counter electrode, a Ag^+/Ag pseudo-reference electrode with a glass frit, and a 3 mm diameter glassy carbon working electrode. The bulk electrolysis was conducted with flowing inert gas under constant convection and a constant E_{appl} of –1.4 V vs. SCE. The sampling electrolysis experiments were conducted in the customized three-electrode electrochemical reactor with gas inlet/outlet ports as pictured in Figure S1. In a typical experiment, the electrochemical cell contains 100 μM NBT dissolved in the electrolyte solution, 0.1 M $TBAClO_4$ in 1,2-DFB. The working electrode was a Si nanowire array with an average wire length of 15 μm and diameter of 50 nm.[3] The other electrodes included a Pt wire as the counter electrode and a Ag^+/Ag pseudo-reference electrode with a glass frit. The bulk electrolysis was conducted under a constant E_{appl} of –1.4 V vs. SCE. A mixture of CH_4 (Airgas, 99.5%) and house air were introduced into the reactor such that P_{CH4}/P_{air} = 35. In conjunction, control experiments were conducted with flowing N_2 in place of CH_4 at the same constant ratio of 35. For the sampling experiments, 200 μL samples were taken every 3 min, diluted 10-fold with 100 μM NBT solution, and their UV-Vis absorption at 600 nm was measured upon sampling (Figures 3C and 3D). Additionally, samples were taken before and after the 3 h bulk electrolysis with Rh^{III}, CH_4, and NBT, and were analyzed via GC-MS to observe CH_3OH

generation. The reported data are after *iR* correction. Cyclic voltammograms of decamethylferrocene were also conducted to calibrate the potentials of Ag^+/Ag pseudo-reference electrode, based on the reported standard potential of decamethylferrocene (−0.059 V vs. SCE).[8]

D. Determination of $O_2^{\bullet-}$ formation rate $\partial Y_{ROS}/\partial t$. The concentration of $O_2^{\bullet-}$ generated during the sampling electrolysis experiments was quantified through its reaction with NBT via UV-Vis spectroscopy. As the absorption at 600 nm eventually plateaued over time (Figures 3C and 3D), that was taken as the steady-state $O_2^{\bullet-}$ concentration. Thus, the absorption at 600 nm was converted to the amount of $O_2^{\cdot-}$ by way of the experimentally determined ε_{600nm} of 4321 $M^{-1} \cdot cm^{-1}$ and the stoichiometry of the reaction between $O_2^{\bullet-}$ and NBT (2:1 equivalent ratio)[7], before being normalized by the average current. The rate of formation of $O_2^{\bullet-}$ during electrolysis measurements was identified as the slope of the line before the steady-state was reached (0–30 min). The slope of equivalent trials was averaged and ultimately, this value was taken as the rate of formation.

E. Karl Fischer titration. The amount of water impurities in neat 1,2-DFB was quantified via a Mettler Toledo C20 Coulometric Karl Fischer Titrator. Neat 1,2-DFB was injected into the Karl Fischer solution at volumes of 0.3, 0.5 and 1.0 mL. Each volume injected was replicated 6 times to minimize the standard deviation and overall error. The average water content in the 1,2-DFB was found to be 92.2±5.7 ppm (or 5.1±0.3 mM).

Section 4. Reactivity between Rh^{III}–CH_3 and ROS

Stoichiometric reactions between Rh^{III}–CH_3 and different ROS species were performed and monitored by ^1H NMR. In one example, 0.25 mM Rh^{III}–CH_3 and 0.25 mM *t*-butyl hydroperoxide were mixed in 1,2-DFB for 6 hrs at room temperature. The ^1H NMR spectra in C_6D_6 indicate the formation of CH_3OH at the expense of the axial methyl group of Rh^{III}–CH_3. Similar results were also obtained with cumene hydroperoxide in decane, 2-(1- hydroperoxy-1-methoxyethyl)-5-methylcyclohexan-1-ol in 1,2-DFB, and KO_2 in "wet" 1,2-DFB. Reactions between 1 mM Rh^{III}–CH_3 and KO_2 in strictly dry 1,2-DFB did not yield any observable formation of CH_3OH up to 48 h at room temperature .

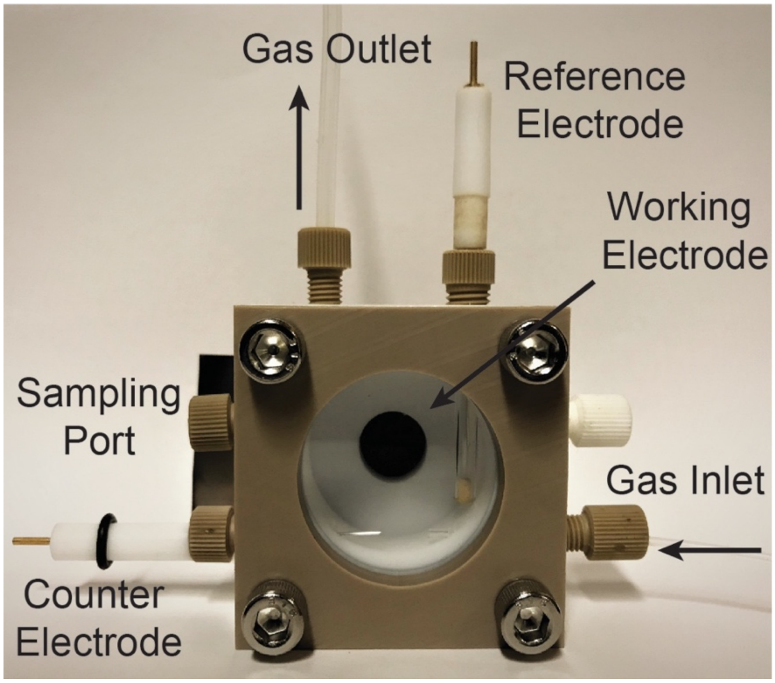

Figure S1. Photograph of the customized electrochemical reactor used for bulk electrolysis.

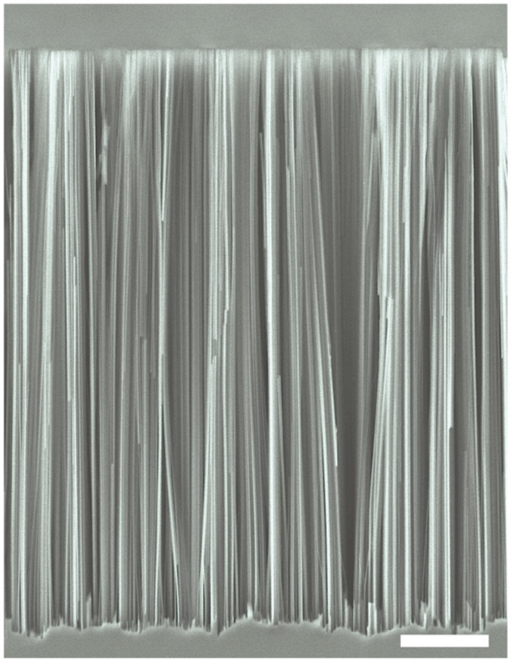

Figure S2. Scanning electron microscopy image of silicon nanowire array with length of 15 μm and diameter of 50 nm. Scale bar, 2 μm.

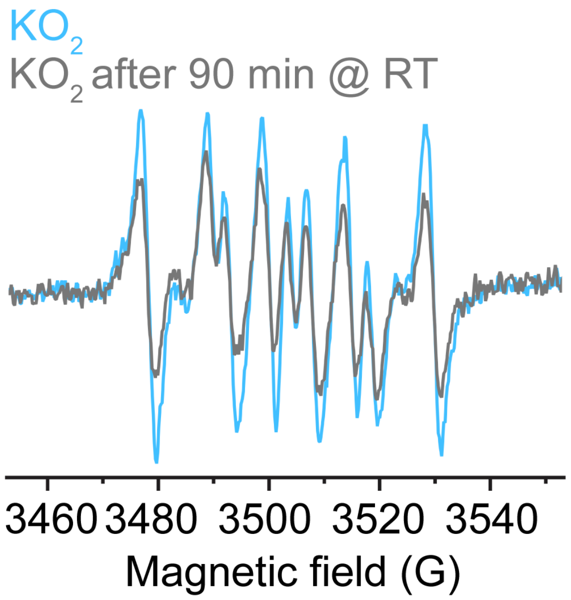

Figure S3. Electron paramagnetic resonance spectra depicting the adducts formed upon the reaction of 50 mM DMPO (5,5-dimethyl-1-pyrroline N-oxide) and 1 mM KO_2 (potassium dioxide) after immediate thawing (light blue trace) and the same reaction mixture after 90 min at room temperature, RT (silver trace); 0.1 M $TBAClO_4$ (tetrabutylammonium perchlorate) in 1,2-DFB (1,2 difluorobenzene).

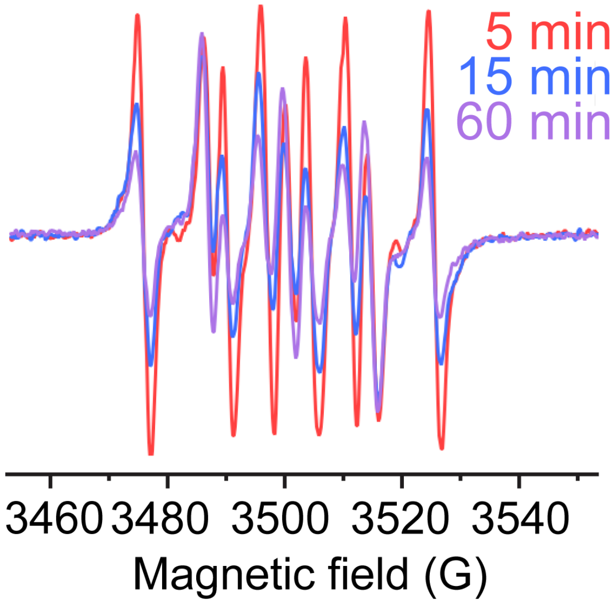

Figure S4. Electron paramagnetic resonance spectra depicting the adducts formed upon the addition of 50 mM DMPO (5,5-dimethyl-1-pyrroline N-oxide) to the constant potential electrolysis; samples were taken 5 min (red curve), 15 min (blue curve), and 60 min (purple curve) after the addition of 50 mM DMPO; 1 mM Rh^{III} in the electrolyte solution, 0.1 M $TBAClO_4$ (tetrabutylammonium perchlorate) in 1,2-DFB (1,2 difluorobenzene); constant reduction potential of –1.4 V vs. SCE was applied to the Si nanowire working electrode; a mixture of CH_4 and air were flowed into the customized electrochemical reactor (Figure S1) at a defined ratio of 35 ($P_{CH4}/P_{air} = 35$) under ambient pressure.

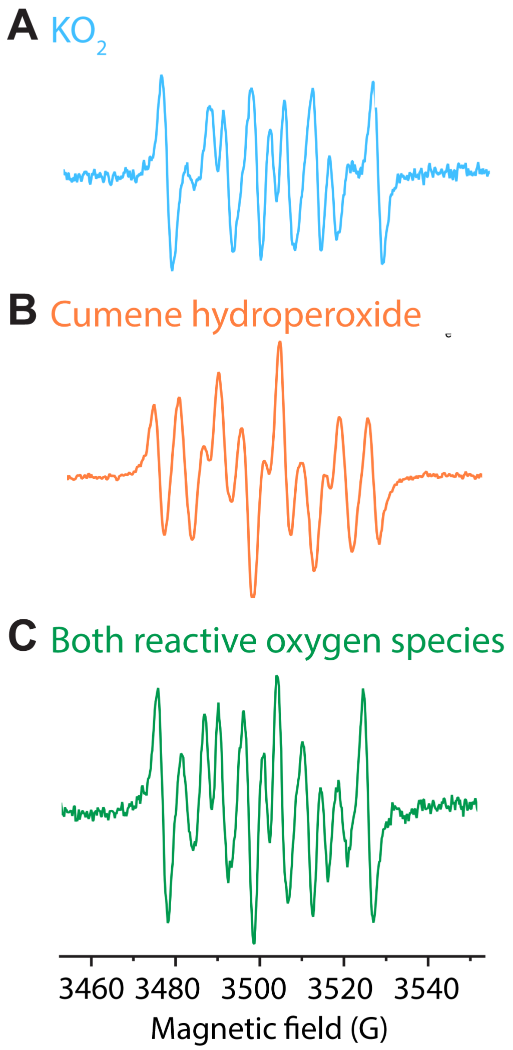

Figure S5. Electron paramagnetic resonance spectra depicting the adducts formed upon the reaction of 50 mM DMPO (5,5-dimethyl-1-pyrroline N-oxide) and (**A**) 500 μM KO_2 (potassium dioxide) (**B**) 500 μM cumene hydroperoxide (2-hydroperoxypropan-2-ylbenzene) and (**C**) both 500 μM KO_2 and 500 μM cumene hydroperoxide in the electrolyte solution, 0.1 M $TBAClO_4$ (tetrabutylammonium perchlorate) in 1,2-DFB (1,2 difluorobenzene).

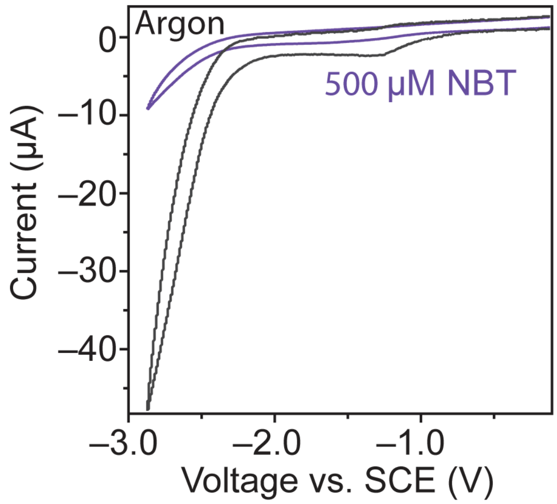

Figure S6. Cyclic voltammograms on a glassy carbon electrode under argon environment. Dark gray trace, blank without the addition of NBT (nitroblue tetrazolium); purple trace, 500 μM NBT; 0.1 M $TBAClO_4$ (tetrabutylammonium perchlorate) in 1,2-DFB (1,2 difluorobenzene), 100 mV/s.

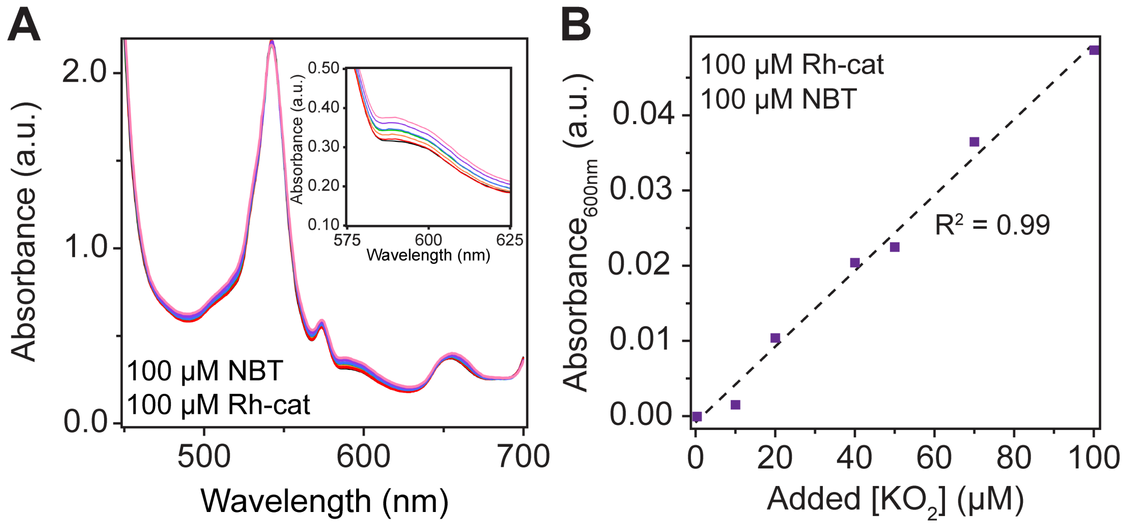

Figure S7. (**A**) Ultraviolet-visible absorption spectrum monitoring the reaction product of 100 μM NBT (nitroblue tetrazolium) with varying concentrations of added KO_2 (potassium dioxide) in the presence of 100 μM Rh-cat (Rh^{III}, (TMP)Rh–I); 0 μM KO_2 (black), 10 μM KO_2 (red), 20 μM KO_2 (orange), 40 μM KO_2 (green), 50 μM KO_2 (blue), 70 μM KO_2 (purple), 100 μM KO_2 (pink). (**B**) Beer's Law curve illustrating the reaction product at 600 nm of 100 μM NBT as a function of KO_2 concentration with 100 μM Rh-cat.

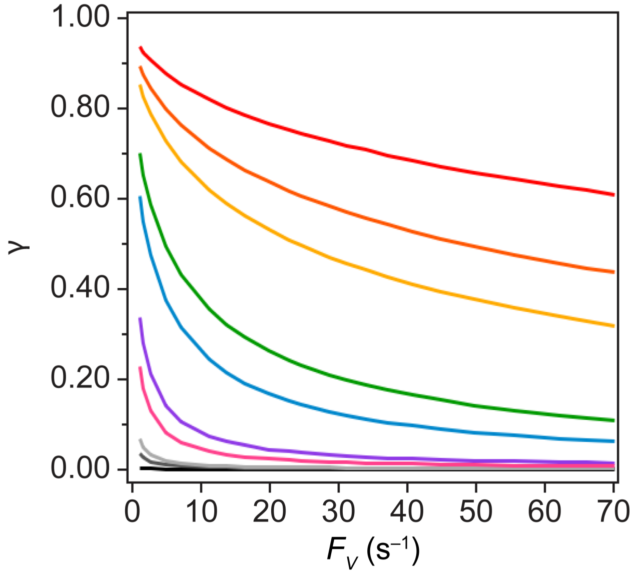

Figure S8. Derived values of γ as a function of F_V for an array of k_2 values 0.132 $L^2 \cdot mol^{-2} \cdot s^{-1}$, (black), the experimentally determined value in the bulk solution[5], 5 $L^2 \cdot mol^{-2} \cdot s^{-1}$ (gray), 10 $L^2 \cdot mol^{-2} \cdot s^{-1}$ (silver), 50 $L^2 \cdot mol^{-2} \cdot s^{-1}$ (pink), 100 $L^2 \cdot mol^{-2} \cdot s^{-1}$ (purple), 500 $L^2 \cdot mol^{-2} \cdot s^{-1}$ (blue), 1×10^3 $L^2 \cdot mol^{-2} \cdot s^{-1}$ (green), 5×10^3 $L^2 \cdot mol^{-2} \cdot s^{-1}$ (light orange), 1×10^4 $L^2 \cdot mol^{-2} \cdot s^{-1}$ (dark orange) and the experimentally determined value within the nanowire 2.9 $\times 10^4$ $L^2 \cdot mol^{-2} \cdot s^{-1}$ (red).

Additional references

1. Tsitkov, S.; Hess, H., Design Principles for a Compartmentalized Enzyme Cascade Reaction. *ACS Catal.* **2019,** *9*, 2432−2439.
2. Berg, H. C., Diffusion Macroscopic Theory. In *Random Walks in Biology*, REV - Revised ed.; Princeton University Press: 1993; pp 17−36.
3. Natinsky, B. S.; Lu, S.; Copeland, E. D.; Quintana, J. C.; Liu, C., Solution Catalytic Cycle of Incompatible Steps for Ambient Air Oxidation of Methane to Methanol. *ACS Cent. Sci.* **2019,** *5*, 1584−1590.
4. Cui; Wayland, B. B., Superoxo, Peroxo, and Hydroperoxo Complexes Formed from Reactions of Rhodium Porphyrins with Dioxygen: Thermodynamics and Kinetics. *J. Am. Chem. Soc.* **2006,** *128*, 10350−10351.
5. Wayland, B. B.; Ba, S.; Sherry, A. E., Activation of methane and toluene by rhodium(II) porphyrin complexes. *J. Am. Chem. Soc.* **1991,** *113*, 5305−5311.
6. Huang, Z.; Geyer, N.; Werner, P.; de Boor, J.; Gösele, U., Metal-Assisted Chemical Etching of Silicon: A Review. *Adv. Mater.* **2011,** *23*, 285−308.
7. Liu, R.-h.; Fu, S.-y.; Zhan, H.-y.; Lucia, L. A., General Spectroscopic Protocol to Obtain the Concentration of the Superoxide Anion Radical. *Ind. Eng. Chem. Res.* **2009,** *48*, 9331−9334.
8. O'Toole, T. R.; Younathan, J. N.; Sullivan, B. P.; Meyer, T. J., 1,2-Difluorobenzene: a relatively inert and noncoordinating solvent for electrochemical studies on transition-metal complexes. *Inorg. Chem.* **1989,** *28*, 3923−3926.
